# Combined Ablation and Resection (CARe) as an Effective Parenchymal Sparing Treatment for Extensive Colorectal Liver Metastases

**DOI:** 10.1371/journal.pone.0114404

**Published:** 2014-12-08

**Authors:** Serge Evrard, Graeme Poston, Peter Kissmeyer-Nielsen, Abou Diallo, Grégoire Desolneux, Véronique Brouste, Caroline Lalet, Frank Mortensen, Stefan Stättner, Stephen Fenwick, Hassan Malik, Ioannis Konstantinidis, Ronald DeMatteo, Michael D'Angelica, Peter Allen, William Jarnagin, Simone Mathoulin-Pelissier, Yuman Fong

**Affiliations:** 1 Digestive Tumours Unit, Institut Bergonié, Bordeaux, France; 2 University of Bordeaux Segalen, Bordeaux, France; 3 Department of Hepatobiliary Surgery, North Western Hepatobiliary Centre, Aintree University Hospitals, Foundation Trust, Liverpool L9 7AL, United Kingdom; 4 Department of Surgery, Aarhus University Hospital, Århus C, Denmark; 5 Clinical and Epidemiological Research Unit, Institut Bergonié, Bordeaux, France; 6 Department of General Surgery, HPB Unit, Paracelsus Private Medical University, Salzburg, Austria; 7 Department of Surgery, Memorial Sloan-Kettering Cancer Center, New York, New York, United States of America; 8 INSERM ISPED, Centre INSERM U897-Epidemiologie-Biostatistique, Clinical Epidemiology and Clinical Investigation Centre CIC1401, Bordeaux, France; Hokkaido University, Japan

## Abstract

**Background:**

Combined intra-operative ablation and resection (CARe) is proposed to treat extensive colorectal liver metastases (CLM). This multicenter study was conducted to evaluate overall survival (OS), local recurrence-free survival (LRFS), hepatic recurrence-free survival (HRFS) and progression-free survival (PFS), to identify factors associated with survival, and to report complications.

**Materials and Methods:**

Four centers combined retropectively their clinical experiences regarding CLM treated by CARe. CLM characteristics, pre- and post-operative chemotherapy regimens, surgical procedures, complications and survivals were analyzed.

**Results:**

Of the 288 patients who received CARe, 210 (73%) had synchronous and 255 (88%) had bilateral CLM. Twenty-two patients (8%) had extrahepatic disease. Median follow-up was 3.17 years (95%CI 2.83–4.08). Median OS was 3.33 years (95%CI 3.08–4.17) and 5-year OS was 37% (95%CI 29–45). One- and 5-year LRFS from ablated lesions were 87.9% (95%CI 83.3–91.2) and 78.0% (95%CI 71–83), respectively. Median HRFS and PFS were 14 months (95%CI 11–18) and 9 months (95%CI 8–11), respectively. One hundred patients experienced complications: 29 grade I, 68 grade II–III–IV, and three deaths. In the multivariate models adjusted for center, the occurrence of complications was confirmed as a major independent factor associated with 3-year OS (HR 1.80; *P* = 0.008). Five-year OS was 25.6% (95%CI 14.9–37.6) for patients with complications and 45% (95%CI 33.3–53.4) for patients without.

**Conclusions:**

Recent strategies facing advanced CLM include non-anatomic resections, portal-induced hypertrophy of the future remnant liver and aggressive medical preoperative treatments. CARe has the qualities of an approach that allows effective tumor clearance while maintaining good tolerance for the patient.

## Introduction

Approximately 80–85% of patients with colorectal liver metastases (CLM) will not be suitable for upfront resection [1]. However, current chemotherapy regimens can convert between 19% [2] to 28% [3] of initially considered unresectable CLM to resectable CLM. For patients requiring protracted regimens to achieve resectability, liver parenchymal damage occurs, necessitating a more conservative and better tolerated tumor eradication strategy than radical resection. [4]

Thermal ablation is a parenchymal-sparing treatment for hepatic malignancies that is particularly effective for small tumors. [5] For hepatocellular carcinoma, randomized trial data show that thermal ablation may be equivalent to resection and well tolerated. [6] For CLM, ablation with resection is increasingly used as a parenchymal-sparing strategy that combines effective ablative eradication of small tumors [7] with resection of large tumors where thermal ablation is less effective. The Beaujon Hospital report concluded that patients who responded only after 12 chemotherapy cycles [4] should undergo conservative strategies including repeated resection, ablation or intra-arterial chemotherapy instead of extensive radical surgery. In the Paul Brousse Hospital series, local treatments, including radiofrequency ablation or cryotherapy, in combination with hepatectomy after downsizing chemotherapy, were introduced for 21% of patients with initially unresectable CLM with good results and reduced morbidity. [2]

The present study design draws upon two recent prospective trials: EORTC 40004, in which the combination of ablation (with or without resection) plus downsizing chemotherapy resulted in 30-month OS of 62% [8]; and a phase II study in which patients undergoing ablation (with or without resection) achieved a 5-year OS of 43% [9]. The present study was conducted on a larger multicenter basis to validate these recent estimates of OS for patients with CLM treatable surgically only by combining resection with ablation. Secondary objectives were to estimate local recurrence-free survival (LRFS), hepatic and/or healthy liver lesion recurrence-free survival (HRFS) and progression-free survival (PFS), to identify factors associated with survival, and to analyze postoperative complications and their potential impact on outcome.

## Materials and Methods

### Patients

Patient records were retrospectively identified from prospectively-maintained databases at each participating institution (Aarhus University Hospital, Denmark; Aintree University Hospital, Liverpool, UK; Institut Bergonié, Bordeaux, France; and Memorial Sloan Kettering Cancer Center, New York, USA).

Patients treated between January 2001 and December 2011 with resection and ablation (radiofrequency and/or microwaves) achieving a complete (R0) resection of all metastases (including extra-hepatic, if any), and who were 18 years or older were included for the analyses on a consecutive basis.

At each center, the overarching strategy was a parenchyma-saving approach. The decision for ablation was made by the operating surgeon, who decided which lesion(s) would be ablated and which would be resected based on formal criteria for ablation [10] such as maximum size, location with respect to the biliary tract and dispersion in the parenchymal tissues. Criteria for ablation within each center evolved over time, initially including only small deep lesions, to include bilateral lesions [7], and lastly, lesions close to parahepatic veins [11]. Patients previously treated with percutaneous ablation of liver metastases were excluded. This retrospective study was approved by the “College de Recherche Clinique” at Institut Bergonié and the Liverpool Local Research Ethics Committee for Aintree University Hospital. The need for informed consent was waived by the Institutional Review Board for the Memorial Sloan Kettering Cancer Center and Aarhus University Hospital did not need approval by the Danish Medicines Agency/Ethics Committee to perform this kind of study. The international data collection was approved by the Advisory Committee on the treatment of information in the field of health research (Comité consultatif sur le traitement de l'information en matière de recherche dans le domaine de la santé, CCTIRS). Since this was a retrospective study previous consent was not obtained from the patients, and records were anonymized and de-identified prior to analysis.

### Multidisciplinary management

Patient cases were generally discussed at multidisciplinary team (MDT) meetings involving surgeons, oncologists and radiologists. Limited, resectable extrahepatic disease was not necessarily an exclusion criterion. The need for chemotherapy was discussed pre- and post- operatively by the MDT for each patient, as well as the use of a hepatic artery infusion pump.

Response to chemotherapy was evaluated per lesion after every four cycles by CT scan and/or magnetic resonance imaging (MRI). Patients responding to chemotherapy were reconsidered for surgery if resection plus ablation could clear intra- and extra-hepatic metastases. If the volume of the liver to be preserved was considered insufficient, preoperative portal vein embolization (PVE) was performed.

### Surgical treatment

Surgery began with a thorough laparotomy to detect any other intra-abdominal disease. After liver mobilization, an intra-operative ultrasound examination (IOUS) of the liver was carried out. The final technical decision regarding the surgical procedure was taken after the IOUS based on the following: possible discovery of new lesions, hepatic volume, liver-related chemotoxicity and the need to resect the primary colorectal cancer. The decision to perform a two-stage procedure was taken in selected cases, for example, by initially clearing the future liver remnant of disease and carrying out a contralateral PVE.

Resections included major and minor hepatectomies, and anatomical and non-anatomical resections. In most cases, intermittent clamping of the portal pedicle was performed. Wedge and segmental resections were guided by IOUS.

Ablation was conducted by the surgeon under IOUS guidance, following previously described and validated techniques. [10] While in the early years of the study period, patients were treated by radiofrequency ablation, microwave ablation was subsequently used with greater frequency. Both one-shot and overlapping strategies of needle positioning were used, depending on the size of the target.

Extrahepatic disease (e.g. lymph nodes) was resected during the same procedure when complete clearance was possible. Resectable lung metastases were generally treated 2–3 months later.

### Post-operative management

Post-operative complications within 30 days were recorded according to the Clavien-Dindo grading system [12]. Patients were followed-up by serum tumor markers (carcinoembryonic antigen and CA-19.9) and CT scan from time of liver resection every 4 to 6 months, depending on the center. Indications for post-operative chemotherapy were discussed in a post-operative MDT meeting.

### Statistical methods

Frequencies and percentages are used to describe qualitative variables, and mean and standard deviations for quantitative variables. Events considered for OS were death due to any cause, with surviving patients censored at the date of last news. OS duration comprised the time between surgery and the event or censoring. LRFS was considered as the time between surgery and recurrence at the site of ablation. Local recurrence was defined as contrast-enhancement surrounding the ablation scar identified on CT scan at follow-up visits. Patients were censored if they had no recurrence of ablated lesions on the date of last news, or death without recurrence at an ablation site. HRFS was time from surgery to date of censoring or event defined as hepatic recurrence. Patients with no hepatic recurrence were censored at the date of last news or date of death if they died without hepatic recurrence. PFS was considered as time from surgery to date of censoring or event defined as any progression. Patients with no progression were censored at the date of last follow up or date of death, if they died without progression. Median follow-up was calculated via the reverse Kaplan-Meier method. [13] Curves for OS, local (ablated lesion) recurrence-free (LRFS), HRFS, and PFS are estimated by the Kaplan-Meier method [14]. Recurrence data was missing for three patients and progression-free survival for two; they were therefore excluded from the LRFS and PFS analyses, respectively.

For univariate and multivariate survival analyses data were censored at 3 years. The following factors were tested for associations with survivals at three years in univariate analyses: age at surgery (>/≤60 years); gender; bilateral hepatic metastases (yes/no); synchronous metastases (yes/no); complications (yes/no); pre-operative chemotherapy (yes/no); targeted pre-operative therapy (yes/no); number of pre-operative chemotherapy lines (0, 1, 2 or 3); American Society of Anesthesiologists (ASA) score (1, 2, 3); number of metastases resected (</≧2); number of metastases treated by intra-operative ablation (>/≤2); maximum size of lesions (<1 cm, 1–3 cm, ≧3 cm), and the existence of extra hepatic metastases (yes/no). Due to the exploratory nature of the study and to avoid omitting important variables we used a *P*<.20 value as a threshold for inclusion in a stepwise ascending multivariate manual Cox model adjusted for center. A significant association with survival was considered at *P*<0.05. Patients with missing values (less than 10%) were excluded from the multivariate analysis.

All data were performed in SAS, v9.2 (Cary, NC).

## Results

### Patients

A total of 288 patients were included (27, 53, 70, 138 patients included per center). Table 1 summarizes patient characteristics. Over half the patients were male (62%), with a median age of 61 years. The median number of metastases was 5 (range: 2 to 21). Median number of tumors resected was 2 (range: 1 to 19) and ablated 2 (range: 1 to 12). The median size of the largest ablated lesion per patient was 10 mm (range: 3 to 50). Extra-hepatic disease was resected in 22 patients (8%). Portal vein obliteration was necessary in 28 patients (10%).

**Table 1:** Patient characteristics for patients treated by intraoperative ablation for liver metastases (*N* = 288).

Characteristics *N* (%)		
Median age at surgery (range)	61 (26–87)	
Sex		
Male	180	(62.5)
Female	108	(37.5)
Synchronicity of metastases		
Synchronous metastases	210	(72.9)
Metachronous metastases	76	(26.4)
Missing	2	(0.7)
Bilateral hepatic metastases	255	(88.5)
Extra hepatic disease	22	(7.6)
Size of largest liver lesion		
<1 cm	33	(11.5)
1–3 cm	200	(69.4)
≧3 cm	30	(10.4)
Missing	25	(8.7)
Pre-operative chemotherapy for liver metastases	232	(81.0)
1 line	178	(76.7)
2 lines	37	(15.9)
3 lines	10	(4.3)
Missing	7	(3.0)
Pre-operative targeted therapy	129	(44.8)
Missing	10	(3.5)
ASA* score		
1	25	(8.6)
2	184	(64.0)
3	75	(26.0)
Missing	4	(1.4)
Complications (Clavien-Dindo)		
No	188	(65.3)
Yes	100	(34.7)
Grade I	29	(29.0)
Grade II	19	(19.0)
Grade III	38	(38.0)
Grade IV	11	(11.0)
Grade V	3	(3.0)
Post-operative chemotherapy	191	(66.3)
1 line	104	(54.5)
2 lines	46	(24.1)
3 lines	4	(21.5)

**Abbreviations:** *N* =  number of patients, *ASA =  American Society of Anesthesiologists.

### Complications

Overall 30-day post-operative mortality was 1%, with three deaths due to gastrointestinal bleeding and septic shock (1), and liver failure (2). One hundred patients (35%) experienced complications (Table 1) and eleven required reoperation.

### Survival analyses

#### Overall survival (OS)

**Figure 1:**
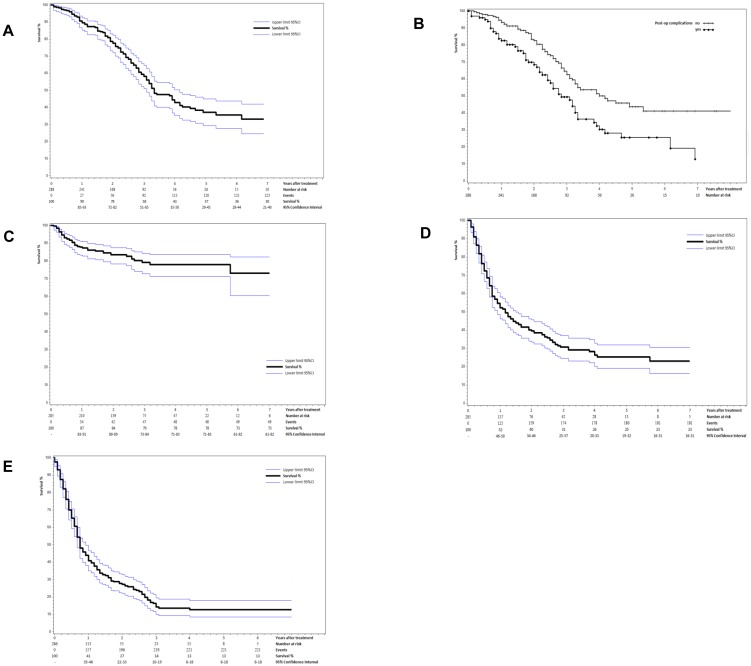
Survival curves and for patients with liver metastases treated by resection combined with intra-operative ablation (IOA). **A** Overall survival (OS) and 95% confidence intervals (CI). **B** OS according to whether complications occurred or not during resection combined with IOA. **C** Local recurrence-free survival (LRFS) and 95%CI for patients treated by surgery and ablation for colorectal liver metastases (CLM) (*N* = 285). **D** Hepatic recurrence-free survival (HRFS) and 95%CI for patients treated by surgery and ablation for CLM (*N* = 285). E Progression-free survival (PFS) and 95%CI for patients treated by surgery and ablation for CLM (*N* = 285).

Median follow-up was 3.17 years (95%CI 2.83–4.08) and median OS was 3.33 years (95%CI 3.08–4.17). Five-year OS was 37% (95%CI 29–45) (Fig. 1A). One hundred twenty four (43%) deaths were observed, including 93 (32%) within three years. In univariate analyses, complications (*P* = 0.009), extra hepatic disease (*P*<0.001) and maximum lesion size ≧1 cm (*P* = 0.106) were the only factors associated with lower 3-year survival. In the multivariate model adjusted for center, the occurrence of complications (HR 1.80; 95%CI 1.16–2.76; *P* = 0.008) and the existence of extra hepatic metastases (HR 2.01; 95%CI 1.03–3.94; *P* = 0.041) were confirmed as independent factors associated with lower 3-year OS (Table 2). Five-year OS was 25.6% (95%CI 14.9–37.6) for patients with complications and 45% (95%CI 33.3–53.4) for patients without (Fig. 1B).

**Table 2:** Factors associated with survival over three years after Combined Ablation and Resection (CARe) for Extensive Colorectal Liver Metastases.

	Hazard Ratio	95% confidence interval	*P*
**Overall survival at three years**			
Presence of complications			
Yes	1.80	1.16–2.76	0.008
No	1 (Reference)		
Existence of extra hepatic metastases			
Yes	2.01	1.03–3.94	0.041
No	1 (Reference)		
**Local recurrence-free survival at three years**			
Maximum size of the lesion			
<1 cm,	1 (Reference)		0.071
1–3 cm	2.88	0.68–12.13	0.151
≧3 cm	5.58	1.14–27.23	0.034
**Hepatic recurrence-free survival at three years**			
Bilateral liver metastases			
Yes	2.10	1.12–3.89	0.019
No	1 (Reference)		
Presence of complications			
Yes	1.40	1.03–1.90	0.035
No	1 (Reference)		
Number of metastases treated by intra-operative ablation			
≧2	1.40	1.03–2.00	0.033
<2	1 (Reference)		
**Progression-free survival at three years**			
Synchronous liver metastases			
Yes	1.57	1.12–2.19	0.008
No	1 (Reference)		
Presence of complications			
Yes	1.40	1.05–1.86	0.022
No	1 (Reference)		
Pre-operative targeted therapy			
Yes	1.41	1.05–1.88	0.021
No	1 (Reference)		

#### Local recurrence-free survival (LRFS)

Local recurrence of ablated lesions was observed in 49 patients (17%), including 47 within three years (16%). Median LRFS was not reached. One- and 5-year LRFS from ablated lesions were 87.9% (95%CI 83.3 to 91.2) and 78% (95%CI 71–83) respectively (Fig. 1C). The initial size of the recurrent ablated lesion was available for 29 of the 49 lesions, the corresponding mean lesion size was 19.4 mm (SD: 11.6). In the univariate analyses the following factors were associated with higher risk of 3-year local failure on the ablation site: bilateral hepatic metastases (P = 0.130); synchronous metastases (P = 0.160);>2 metastases treated by ablation (P = 0.079); and maximum lesion size (P = 0.045). In the multivariate model adjusted for center, maximum lesion size in three categories (<1 cm, 1–3 cm,>3 cm) approached statistical significance (P = 0.070). Only lesions>3 cm showed a lower 3-year LRFS than lesions <1 cm (HR 5.58; 95%CI 1.14–27.22, P = 0.034) (Table 2).

#### Hepatic recurrence-free survival (HRFS)

Liver recurrence was observed (on treated lesions or healthy remnant liver) in 182 (64%) of 285 patients (data missing for three patients), including 175 (61%) within three years. Median HRFS was 14 months (95%CI 11–18) and HRFS at five years was 25% (95%CI 19–32) (Fig. 1D). Factors associated with lower 3-year HRFS included: presence of bilateral hepatic metastases (P = 0.016); perioperative complications (P = 0.078); targeted pre-operative chemotherapy (P = 0.086); number of metastases resected (P = 0.170); number of metastases treated by radiofrequency (P = 0.033); and maximum lesion size (P = 0.170). In the multivariate model adjusted for center, the following factors were independently associated with lower 3-year HRFS: bilateral liver metastases (HR 2.10; 95%CI 1.12–3.89; P = 0.019); presence of complications (HR 1.40; 95%CI 1.03–1.90; P = 0.035); and ≧ two metastases treated by intra-operative ablation (HR 1.40; 95%CI 1.03–2.00; P = 0.033) (Table 2).

#### Progression-free survival (PFS)

Median PFS was 9 months (95%CI, 8–11) and 5-year PFS was 13% (95%CI 8–18) (Fig. 1E). In total, disease progression was observed in 221 patients (77%), including 219 (77%) within the first three years. Factors associated with lower 3-year PFS included: bilateral hepatic metastases (P = 0.036); synchronous metastases (P = 0.019); complications (P = 0.049); pre-operative chemotherapy (P = 0.047); pre-operative targeted therapy (P = 0.009); higher number of pre-operative chemotherapy lines (P = 0.068); number of metastases treated by ablation (P = 0.023); and maximum lesion size (P = 0.014). In the multivariate model adjusted for center, the following factors were independently associated with lower 3-year PFS: synchronous liver metastases (HR 1.57; 95%CI 1.12–2.19; P = 0.008); presence of complications (HR 1.40; 95%CI 1.05–1.86; P = 0.022); and pre-operative targeted therapy (HR 1.41; 95%CI 1.05–1.88; P = 0.021) (Table 2).

## Discussion

The indications for resection of colorectal liver metastases have greatly evolved in recent years [15]. Bilateral, synchronous, or disease involving extrahepatic sites were previously considered as criteria for unresectability. Downsizing chemotherapy has increased the number of technically resectable patients, but it causes parenchymal damage and higher post-operative complication rates [16], [17]. As a consequence, caution is required in resection approaches and parenchymal conservation should be considered. The combined ablation and resection (CARe) approach, taking advantage of the ability of liver-sparing ablations to destroy small tumors, and to best use resections for removal of large tumors, is a rational de-escalating approach compared to more extensive hepatectomy [4]. Non-anatomical resection was long considered to be a transgression of the orthodox liver surgery based on vascular rules inherited from transplant surgery [18]. It was later accepted [19] and lastly brought to its peak by Torzilli et al [20] most notably to allow iterative procedures. Ablation is a pragmatic non-anatomical concept, with a high propensity to spare healthy parenchyma.

There have been prior reports supporting CARe as a rational approach. In the Beaujon Hospital series, patients with initially unresectable CLM without extrahepatic diseases responding to chemotherapy had a 5-year PFS of 13% and a 5-year OS of 40% after major hepatic resection. Mortality, however, was high at 10%, and margins were involved in 39% of the cases. Patients requiring more than 12 cycles of chemotherapy to achieve resectability had more post-operative complications, a 3-year DFS of 0% and a 5–year OS of less than 30% [4].

A retrospective study by Karanicolas et al showed that even with poorer prognosis and higher clinical risk factors, patients undergoing an ablation plus resection approach achieved nearly the same 5-year survival (56%) as those undergoing resection alone (49%) [7]. Further, the combined approach translated into improved post-operative outcomes, confirming the safety of the procedure.

Comparing the results of different series of unresectable CLM is problematic due to the heterogeneous patient population and the variable definition of resectability [3] (Table 3). Three-year PFS ranges between 10% [9] and 28% [8] and 5-year OS between 33% [2] and 56% [7], depending on factors such as the median number of CLM, synchronous or metachronous LM, and the presence of extra-hepatic disease. The effect of the latter on survival has already been reported [2], and is also reflected in the present study: 3-year OS doubles in the absence of extra-hepatic disease (30% vs. 60%). Indeed, there is biological evidence that disease confined to only one organ or oligometastatic with limited metastatic capacity has a better associated prognosis [21]. Our PFS and OS compared well to those of the Paul Brousse series [2], where the inclusion criteria were similar to those in the present study (particularly with the addition of some patients with extra-hepatic disease), although ablation was used in only 21% of the treatments. On the other hand, the high 1-year LRFS rate of 89% should free ablation of any incrimination of poor local control abilities.

**Table 3:** Colorectal liver metastases treatment technique and results in different series.

Trial	Study type	Technique	Tumor characteristics			PFS	30-day mortality	5-year OS
			CLM *	CLM characteristics	Extra-hepatic disease			
MSKCC^6^	Retrospective	Resection plus ablation	4	–	no	–	2.1%	56%
CLOCC^2^	Randomized Phase II	Resection plus ablation	4	43% synchronous	no	3-year 28%	1.7%	40%
ARF2003^8^	Phase II	Resection plus ablation	5	84% bilateral	no	3-year 10%	1.9%	43%
Beaujon series^5^	Retrospective	Resection 100% PVO	6	84% synchronous, 78% bilobar	no	3-year 17%	10.3%	40.5%
MD Anderson^17^	Retrospective	Resection plus 3% ablation, 70% PVO	6	80% synchronous, 100% bilobar	no	3-year, 5-year 20%	6.4%	51%
Paul Brousse series^3^	Retrospective	Resection plus 21% ablation, 60% PVO	5	71% synchronous, 76% bilobar	13.5%	5-year 19%	0%	33%
Present study	Retrospective	Resection plus 100% ablation, 10% PVO	5	73% synchronous, 88% bilobar	8%	5-year 13%	1%	37%

*Median; **Abbreviations**: CLM =  colorectal liver metastasis, PVO =  portal vein occlusion; PFS =  progression-free survival, OS =  overall survival

In this analysis, post-operative complications remain a major prognostic factor for 5-year OS, since they reduce drastically patients' chance for OS, from 25.6% with complications to 43.6% without. The negative impact of perioperative morbidity has been demonstrated in several other malignancies, including CLM [22]. While this association has been clearly established, it is unclear whether a direct cause-effect relationship between complications and survival exist. For example do pro-inflammatory processes stimulate tumor progression [23], does time to chemotherapy play a role, or does the poorer survival following complications reflect more aggressive treatment for a greater tumor burden? Post-operative complications also diminish patients' chance to receive adjuvant chemotherapy [4]. CARe is associated with mortality rates between 1 to 4% [7]–[9] compared to 6 to 10% [4], [24] achieved with extensive resection after conversion chemotherapy. Comparing morbidity is more problematic since complications in many resection series were not classified into grades and grade 1 complications were often omitted. By the same criteria, the morbidity rate of 23.6% in this series compares favorably with those of previous studies [4,4].

In order to reduce post-operative complications after liver resection, Tzeng and Vauthey proposed the following guidelines: to reduce the duration of pre-operative chemotherapy; to avoid combined resections of liver and primary tumor in cases of synchronous disease; to consider two-stage procedures for bilateral disease; to use PVE to reduce the risk of liver failure; and to favor non-anatomic resections rather than sacrificing healthy parenchyma with formal hemi-hepatectomies [25]. We suggest that CARe be included among the propositions, which diminishes the morbidity associated with major surgical procedures (Table 3).

Both PVE and two-stage procedures are often necessary to treat bilateral advanced cases by resection-only, as reported in several studies (Table 3). Increasing the use of ablation from 0% [4] to 100% (present study) results in a reduction in the use of PVE from 100% [4] to 10% and a reduction in mortality. It has also been reported that CARe diminishes the use of two-stage procedures [20] and even overall costs [26].

Limitations of this study include missing data due to the retrospective analysis and the disparities inherent to practices of different surgeons. Nevertheless, this exploratory study based on prospectively recorded data, to our knowledge the largest of its kind, is pivotal in understanding the role of CARe as part of a multidisciplinary approach.

Despite the drawback of some hepatic toxicity induced, conversion chemotherapy has changed the long-term outlook of unresectable and borderline resectable cases. It is commonplace for the liver surgeon to consider hepatectomy after the patient has already been subjected to triplet drug regimens with targeted therapies, or after intra-arterial chemotherapy [27]. For many of these patients, CARe seems to allow an effective and safe treatment option that activates two strategies in one: a de-escalation of normal parenchyma and an escalation on metastases that allows targeted and iterative surgeries. Facing advanced colorectal liver metastases, a parenchyma-saving approach is key. Technical implementations require a high-level of expertise in IOUS for the liver surgeon. Along with increasing reports of an extension of the indications for RFA [7], [28], this report marks the end of a taboo indicating that RFA is now approved to complement resection or, in selected cases, to replace it.
